# Recent Advancements towards Sustainability in Rotomoulding

**DOI:** 10.3390/ma17112607

**Published:** 2024-05-28

**Authors:** Jake Kelly-Walley, Peter Martin, Zaida Ortega, Louise Pick, Mark McCourt

**Affiliations:** 1 Matrix Polymers, Unit 2, Compass Industrial Park, Spindus Road, Speke, Liverpool L24 1YA, UK; 2 School of Mechanical and Aerospace Engineering, Queen’s University Belfast, Ashby Building, Stranmillis Road, Belfast BT9 5AH, UK; p.j.martin@qub.ac.uk (P.M.); l.pick@qub.ac.uk (L.P.); 3 Departamento de Ingeniería de Procesos, Universidad de Las Palmas de Gran Canaria, Edificio de Ingenierías, Campus Universitario de Tafira Baja, 35017 Las Palmas de Gran Canaria, Spain; 4 Polymer Processing Research Centre, Ashby Building, Stranmillis Road, Belfast BT9 5AH, UK; m.mccourt@qub.ac.uk (M.M.)

**Keywords:** rotomoulding, sustainability, sustainable development, polyethylene, recycling, recyclate, natural fibres, biopolymers, simulation, modelling, process development

## Abstract

Rotational moulding is a unique low-shear process used to manufacture hollow parts. The process is an excellent process method for batch processing, minimal waste and stress-free parts. However, the process has drawbacks such as long cycle times, gas dependency and a limited palette of materials relative to other process methods. This review aimed to shed light on the current state-of-the-art research contributing towards sustainability in rotational moulding. The scope of this review broadly assessed all areas of the process such as material development, process adaptations and development, modelling, simulation and contributions towards applications carving a more sustainable society. The PRISMA literature review method was adopted, finding that the majority of publications focus on material development, specifically on the use of waste, fillers, fibres and composites as a way to improve sustainability. Significant focus on biocomposites and natural fibres highlighted the strong research interest, while recyclate studies appeared to be less explored to date. Other research paths are process modification, modelling and simulation, motivated to increase energy efficiency, reduction in scrap and attempts to reduce cycle time with models. An emerging research interest in rotational moulding is the contribution towards the hydrogen economy, particularly type IV hydrogen vessels.

## 1. Introduction

**Figure 1:**
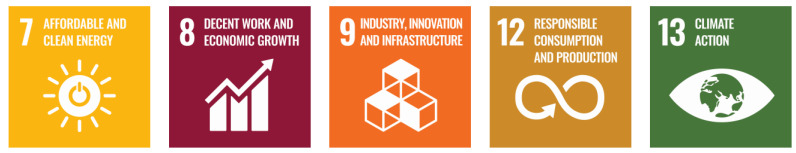
Sustainable development goal (SDG) graphics from the United Nations [2].

Sustainability is now a key consideration within today’s society across all industries, academia, governmental institutions and society. Sustainable development is a core principle and a priority objective of the European Union and, generally, across the globe [1]. Key aspects across relating to sustainability are summarised in the Sustainable Development Goals (SDGs) outlined by the United Nations (UN) as part of Agenda 2030 [2]. In total, there were 17 SDGs identified in 2015 focusing on objectives such as affordable and clean energy (7); decent work and economic growth (8); industry, innovation and infrastructure (9); responsible consumption and production (12); and climate action (13) shown in Figure 1, all of which contribute to 169 targets.

The polymer processing sector has a responsibility as a manufacturing industry to commit to all goals in areas of material design, process modification and scientific understanding. Badurdeen and Jawahir [3] provide a comprehensive definition of sustainable manufacturing as follows: ‘the product, process and systems levels must demonstrate reduced negative environmental impact, offer improved energy and resource efficiency, generate minimum quantity of wastes, provide operational safety and offer improved personnel health, while maintaining and/or improving the product and process quality with overall lifecycle cost benefits’. There is a growing interest in sustainable materials and manufacturing methods across industry and this is reflected in a growing body of the research literature and various industry sector reviews. Authors have emphasised the use of polymers for battery research, increasingly sustainable synthesis routes and polymers from renewable resources like biomass [4,5,6,7]. The review theme continues into the polymer processing field of work, for example, the use of recycled waste and upcycling both plastic and biomass waste, 3D printing developments and applications towards sustainable environmental solutions [8,9,10]. Despite a strong relationship between sustainability and additive manufacture being present in the literature, other studies present in extrusion, composite manufacturing and recycling demonstrate an importance in the relationship of plastics and sustainability [11,12,13]. Reviews align on a common objective to establish the current state of the art in material design, processing and processes towards sustainability for the environment and industry. However, in the case of rotational moulding, there appeared to be no publications reviewing contributions towards sustainability as of today.

**Figure 2:**
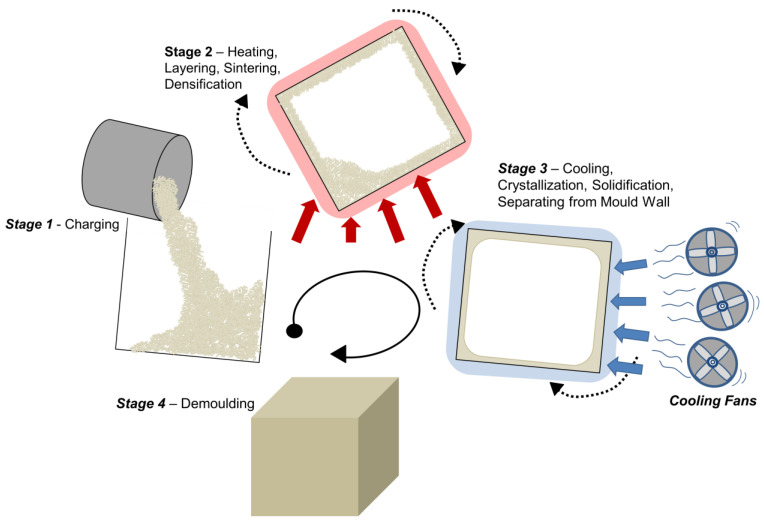
Schematic of the rotational moulding process, simplified to 4 key stages. Red and blue arrows represent heating and cooling respectively. Black arrows indicate biaxial rotation.

The rotational moulding (RM) process offers benefits that cannot be achieved by other polymer processing techniques such as injection or blow moulding. Its manufactured products are stress-free due to no shear processing, it generates minimal waste, it requires inexpensive tooling, it has a high degree of versatility and it is excellent for short production runs [14], all of which justify the selection of RM as a process method for manufacturers and designers [14,15]. Despite these exclusive advantages, the process has some drawbacks related to the limited selection of materials (95% of products are manufactured from PE), greater raw material costs due to grinding and antioxidant addition, long cycle times and current dependency on gas as an energy source [14,16]. Polyethylene dominates the industry due to its relatively low melting temperature and good flow characteristics that complement the low-pressure process; this polyolefin thermoplastic, although used in high volume, has limitations for certain applications [14]. The RM as a polymer processing method is a biaxial process existing fundamentally in four parts with a heating and cooling cycle, as shown in the schematic (Figure 2). It is necessary to understand the basic principles of the process for the context of further review, as many of the limitations for material selection are governed by the nature of the process. Firstly, the tool, which is often fabricated from aluminium or steel, is charged (loaded) with selected material and closed [14]. The tool can be closed by a variety of clamping methods; the tool will still have areas on the tool open for venting during the process cycle. Stage 2 begins where the tool enters an oven and is heated; during the mould rotation, the material starts to heat and layer up on the surface of the tool. This is caused by the tool passing through the powder pool at the bottom of the mould, and as temperature rises, the material starts to become ‘tacky’ or ‘sticky’, allowing it to adhere to the mould wall [14]. As the biaxial rotation and heating continues, layering increases and the powdered material will start to sinter, melt and coalesce, promoting the formation of a polymer melt with trapped air voids [17]. As temperature rises towards the end of the heating cycle, densification occurs, reducing the voids within the polymer melt. The tool will then be removed from the oven once optimum internal air temperature (IAT) is achieved. During this period as demonstrated in Figure 2, stage 3 cooling with fans is commonly used; however, there are other methods that can be selected such as water mist/spray or ambient cooling [14]. The material reaches a peak internal air temperature (PIAT) after removal due before the internal air temperature in the tool starts to decrease leading. The tool and material begin cooling and consequently undergo crystallisation [17]. The product continues to cool before releasing from the tool surface as the material transitions from a viscous polymer melt state to a solid moulded part. Once the tooling has cooled sufficiently and it is safe to do so, the part is de-moulded, concluding the process at stage 4.

RM must adapt and progress towards sustainable development. In rotational moulding, this offers unique challenges and opportunities. For example, high material volume per part (in some cases exceeding 1000 kg) processed in rotational moulding means plenty of opportunity to reduce the net quantity of virgin materials used by utilising waste, renewable sourced materials and furthermore, the adoption of alternatives, which can have a positive impact on the carbon footprint associated with the process and parts. In addition, greater process understanding, modelling and process development can have a considerable impact due to the part volume. A drawback of the process is the long cycle times, which require extended periods of gas consumption, so improving efficiency by exploring alternative energy sources for processing is essential in future work. Sustainability is a fundamental requirement rather than a current trend due to the current climate crisis and challenges around the generation of waste. To maintain substantial growth, continued progress and impactful research, it is crucial to evaluate the current state of the art in response to increased focus. This study aims to address this gap found in reviewing research and outline the position of research in RM towards sustainable development, whether this concerns reuse, renewable materials, greater scientific understanding or reduction in energy usage.

## 2. Methods

This literature review was untaken with a similar systematic approach as outlined by PRISMA [18]. Scopus was selected as the search engine and keywords were used to extract relevant studies from the publication databases from the past decade, 2013–2023. The selection of Scopus was made given its credible source of the scientific literature and the reputation as a well-established, highly regarded platform for research. Such a time period was set to capture the latest state-of-the-art research and most recent contributions to sustainable development, and it has also been a period where this focus has been increasingly topical. Searches were performed in early 2024, including keywords outlined below in Table 1. In addition to the keywords outlined below, “rotational moulding” or “rotomoulding” remained a constant search criterion. 

**Figure 3:**
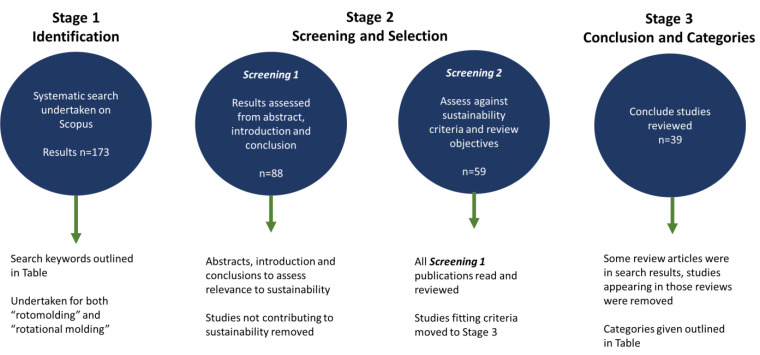
Systematic literature review process from collection to selection.

Figure 3 outlines the process of the literature search and selection of publications for inclusion. The process was constructed from the identification of studies from the literature search (stage 1), then screening and selection, based on abstracts, then full publication assessment (stage 2) before the conclusion and selection (stage 3). Stage 1 collected all research as retrieved from the search while removing any duplicates and those not related to rotational moulding by title. Stage 2 first consisted of reviewing abstracts, and those not relating to sustainability criteria were excluded before stage 2, screening 2. Stage 2, screening 2 publications were read in full, and if the contribution to sustainability was justified, studies proceeded to stage 3 and were reviewed in greater detail for inclusion within this review.

The criteria for selection were an assessment of contributions to sustainability or sustainable development to the rotational moulding research and industry. The definition previously outlined given by Badurdeen and Jawahir et al. [3] on sustainable manufacturing formed the basis for the selection criterion.

**Figure 4:**
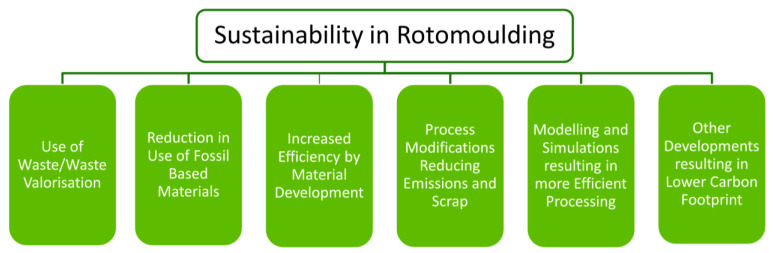
Sustainability criteria in rotational moulding for the purposes of this review.

Contextually, in rotational moulding, this has been defined for the purposes of this research review to contribute towards those outlined in Figure 4. These criteria were used to select relevant studies to be included in this review.

## 3. Results and Discussion

### 3.1. Literature Search Results

**Figure 5:**
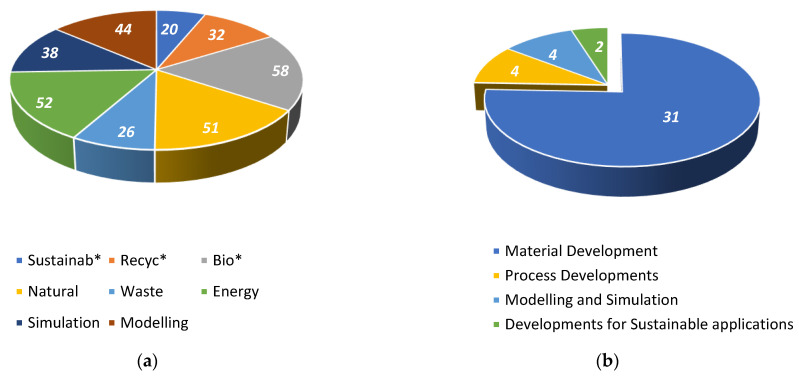
(**a**) Returns from search, population for each search criteria. (**b**) Publications reviewed for each subtitle category.

The total literature search conducted on Scopus resulted in 321 results (sum of Table 1); after removal of duplicates and those not relating to RM by title, 173 studies remained at stage 1. The initial search, prior to stage 1 sorting, showed that the greatest quantity of results were collected from the keywords ‘Energy’ ‘Bio*’ and ‘Natural’, returning 52, 58 and 51 results, respectively. On the other hand, ‘Sustainab*’, ‘Waste’ and ‘Recyc*’ returned the lowest number of results with 20, 26 and 32 results, respectively. The ‘*’ was used to capture multiple suffixes of keywords; for example, ‘Recyc*’ would successfully find studies including Recyclate, Recycling and Recycled. All keywords are outlined in Table 1 and represented graphically in Figure 5a.

Table 2 presents the main categories and sub-categories of the retrieved works; the greatest proportion of the studies were concerning material-based research. Overall, 75% of the publications appearing within this review are categorised as materials research. As a result of the category being so dominant, sub-categories based on publication themes, namely, use of waste and recyclate; natural fibres and fillers; and biopolymers and composites, have been established. The greatest area of research returned was around natural fibres and this was of interest from 2013 to 2023 with steady growth. Use of waste and recycled materials appeared to be an emerging trend from 2018 and has grown in the past 5 years, and studies in this area remain novel to the RM field. This time period does not only relate to polyethylene recyclate but other feedstocks such as tyres, cable waste and biopolymers only appearing in the past 5–6 years. Other categories, such as process research, modelling and simulation and developments for sustainable applications, yielded similar results. Figure 5b showcases the categories of research and quantity of studies in each category.

### 3.2. Material Development

#### 3.2.1. Using Recyclate and Waste Materials

A summary of the publications reviewed and their conclusions are presented in Table 3. A variety of materials have been used from waste or recycled materials throughout the literature.

Chaisrichawla and Dangtungee reported adopting a dry-blended approach, mixing at various compositions [22]. The authors assessed the use of rHDPE (recycled high-density polyethylene) between 0 and 50 wt% melt blended with LLDPE (linear low-density polyethylene). A low MFI ‘blowing process’ recyclate grade was used and good mechanical performance was suggested, and the authors presented small increases in tensile strength with 10 wt% rHDPE compared to neat LLDPE. Young’s modulus was said to significantly increase with the addition of rHDPE. LLDPE also recorded a lower impact strength compared to rHDPE; furthermore, increasing rHDPE content increased impact strength in the LLDPE/rHDPE blends [22]. Shafigullin et al. [28] investigated the use of collected water tanks and water-filled barriers manufactured from a rotational moulding grade. The addition of a plasticising masterbatch, grade PF0010/1-PE, at 4 wt% was described as achieving physical–mechanical performance not inferior to virgin PE. The authors stated that ‘using products of polyethylene recycling ensures high-quality products’ and in this case is suitable for holding tank production [28]. Continuing with studies reporting the use of rPE recyclate, Dou and Rodrigue (2022) studied the use of recycled rHDPE and chemical blowing agent (CBA) azodicarbonamide (AZ) in the RM process [27]. The authors claimed that foam structures were successfully achieved, concluding that a foam structure form of 100% recycled HDPE can be obtained; however, optimisation was suggested. The study also reported reductions in thermal conductivity and increased cell diameter with increasing CBA agent addition when using rHDPE. Within a similar theme, Saifullah et al. [24] also studied foam, more specifically sandwich structures with non-reprocessed and reprocessed materials. Reprocessed material was defined as rotational moulding scrap, purge materials and rejected parts. Low-velocity impact (LVI) and flexure-after-impact (FAI) were of interest for both materials; reports shared that reprocessing sandwich structures produced lower impact performance. Specifically, at 30 J of impact, crack formation was thought to have occurred in reprocessed structures, while it was stated that the virgin structure maintained structural integrity. However, the authors claimed that catastrophic failure was not observed for either material. It was reported that FAI was lower in the reprocessed material, retaining 91% and 66% of the virgin structure strength at 15 J and 30 J, respectively [24]. The authors attributed this to degradation induced by reprocessing, thus influencing the polymeric chain structure and reducing impact strength.

There is a significant variability in the PE recyclate used by authors, ranging from MFI 0.18 to 4 g/10 min (190 °C/2.16 kg) throughout the literature. This appears to be dependent on the source of recyclate and extent of the mechanical recycling process. Pick et al. [19] aimed to provide ‘baseline data’ on polyethylene from recycled rotomoulded tanks and mixed post-consumer (PCR) waste for comparison with virgin polyethylene [22]. A compatibiliser was reportedly used in an attempt to increase miscibility, and in this case, an ionomer of an ethylene acid copolymer was studied. Over a 70% reduction in tensile strength was suggested compared to the virgin performance in the recyclate blends, and improvements in Young’s modulus were reported and thought to be due to the presence of stiffer PP (polypropylene) domains and the presence of fractional melt PEs. The impact performance for recyclate materials was at 0.5 J/mm and 1 J/mm for peak and total impact strength, respectively. The authors believed that the most significant contributing factor was attributed to the presence of PP and lower melt flow PE grades. The large increase in viscosity at low shear rates was also thought to be a significant factor regarding performance when using PCR [19]. Cestari et al. [20] reported a similar study to Pick et al. [19], and in this study, the authors compared various polymer blends of fractional MFI rHDPEs sourced from post-consumer feedstock, bottles pipes and household waste (PCR). Fundamentally, they studied and compared the performance of compression moulded plaques and rotomoulded samples without the presence of a compatibiliser [20]. Improvements in the compression moulding process with the incorporation of recyclate were suggested, whereas the opposite was reported in the rotational moulding process, and there was a specifically significant reduction in impact strength compared to neat MDPE. The authors concluded that the lack of shear in the process of the recyclate materials and incomplete melting was causing a discontinuous structure and hence the reported loss in properties. The researchers suspected that the lower cohesion was also attributed to the 20–30% reductions in the flexural modulus. Use of PCR PP and HDPE from alcohol and mineral water bottles was investigated for use in rotational moulded parts by Ferreira et al. [23]. The authors focused on the use in construction building blocks. Benefits were expected to remove waste from nature, increase the life cycle of the polymer, generate income from waste and use large amounts of waste. The formation of construction blocks was reported as successful, and the authors commented that HDPE (high-density polyethylene) blocks offer ‘excellent material for use in modular construction, in special considering light weighting’. This proof-of-concept study was also said to achieve UL-94 vertical flammability V-0 classification (highest classification under UL-94 vertical standard) with a 5 wt% addition of alumina, thus satisfying and meeting specific construction industry requirements. Cestari et al. [29] also combined a circular economy and rotational moulding by using recyclate in building block applications.

Within the literature reviewed, not only PCR has been reported on, but also some attempts have been made using post-industrial waste/recyclate streams (PIW/PIR). For instance, Díaz et al. focused on the potential reuse of waste cable materials as a filler in rotomolded samples [21]; the objective was to valorise residual cable covers, as the metal wire is extensively recycled and available. Unlike the studies discussed above, this study reported on multi-layer, dual-layer and monolayer approaches. According to the authors, this method allowed the addition of material step by step without the concern of phase separation. However, longer production times and increased complexity of the cycles should also be considered when proposing this layering strategy. The specimens obtained in the work were reported to have a strong reduction in impact, and a linear reduction in Young’s modulus and maximum stress with an increasing fraction of recyclate, demonstrated for both mono- and multilayer structures. The authors attributed this to the inability to melt the thermoset cable waste [21]. Studies assessing the use of valorised PLA cups for rotational moulding were undertaken by Aniśko et al. [25]. Varying particle sizes were reported during the study, ranging from 400 to 1400 µm, and according to Aniśko et al., materials were sieved to assess the influence of particle size. The highest tensile strength was achieved with finer material <400 µm and annealing due to a void volume fraction of 0.54%. Aniśko et al. concluded that results from annealed PLA powder and previously extruded PLA proved the most balanced approach between performance and preparation [25].

Recycled rubber has also been proposed as a filler in the rotational moulding process [26]; particularly, ground tyre rubber (GTR) and LDPE (low-density polyethylene) were blended to produce thermoplastic elastomer materials in RM. Shaker and Rodrigue reported on both dry blending and melt compounding, and the scope was to achieve improved impact strength from the addition of rubber. Materials were prepared at 0, 20, 35 and 50 wt% GTR; in addition, the authors discussed the use of regenerated and non-regenerated GTR. The authors described regenerated rubber as de-vulcanised rubber resulting in a reduced number of crosslinks. The results of pre-treatment on recyclate before blending were discussed and thought to be distinguishable by characterisation. SEM on the RM fractured parts showed a homogenous distribution of GTR in the PE (polyethylene) matrix up to 20 wt% according to the researchers. Shake and Rodrigue reported that non-regenerated rubber had up to a 20% greater flexural modulus than regenerated rubber for both techniques. The authors also suggested that the elongation at break at 20 wt% was above 100%; therefore, good thermoplastic elastomers were achieved and attributed to the elastic nature of the recyclate. Further work was suggested to improve surface pre-treatment [26].

Some of the main challenges with the reviewed literature were high viscosity, porosity in the final article, reduction in mechanical properties and potential requirements to achieve compatibilisation. The importance of minimal contamination was discussed as blend properties were thought to diminish with a polypropylene presence. Melt compounding and dry blending were adopted techniques for material preparation; however, it remains unclear which provided a greater benefit to overall performance due to the absence of studies comparing the same materials like for like, the exception being studies with GTR. There was an absence in addressing the thermal stability directly and the potential improvements this may have on material performance.

#### 3.2.2. Fibres and Fillers

One of the findings from the systematic search was the use of particles in the form of fibres as a filler added to the polymer and used for rotational moulding. This was selected as a category due to the wealth of publications in the rotational moulding literature; however, this trend is not unique and this is a topic of interest for the wider polymer processing field. In total, 10 studies have been assessed under this field. Table 4 provides a summary of the current state of the art retrieved, which shows different plant species used in RM at different loadings. Most research works focus on the use of different grades of PE, due to its wide commercial adoption.

As described by Ortega et al., another route towards more sustainable solutions is using residues or natural fibres [40]. This is thought to be due to the mechanical reinforcement and increase in other mechanical properties, which can be achieved with such organic or inorganic materials, but also complimented by reducing the dependency on using virgin plastics.

From results retrieved, four recent review articles (from 2022 and 2023) exist on this topic. These studies are titled ‘Recent Developments in Inorganic Composites in Rotational Moulding’ by Ortega et al. [40], ‘A review of polymers, fibre additives and fibre treatment techniques used in rotational moulding processing’ by Khanna et al. [41], ‘Effect of Manufacturing Techniques on Mechanical Properties of Natural Fibres Reinforced Composites for Lightweight Products—A Review’ by Sasi Kumar et al. [42], and ‘A comprehensive review to evaluate the consequences of material, additives, and parameterisation in rotational moulding’ by Yadav et al. [43]. These review papers show the relationship and areas of focus between fibres and fillers in RM in detail. In the summary of the review conducted by Khanna et al., the authors reported that the benefits of using natural fibres were the low cost and eco-friendly nature [41]. However, some limitations were identified, i.e., the poor bonding at the interface between the polymer and natural fibre, where a significant effect has been given to pre-treat fibres to reduce their hydrophilic nature and overcome such limitations [41]. There were four main focal areas within these reviews: polymers used in RM, fillers/additives, natural fibres/artificial fibres and pre-treated fibre addition. While Khanna et al. outlines many resins used during rotational moulding research, types of fibres and properties achieved, a large body of the work focuses on pre-treatment. Research on pre-treatments of fibres in rotational moulding was described in that a ‘recommendable piece of work is done to increase the mechanical and thermal properties of fibre-polymer composites’ to date. The authors discussed publications on the following treatments and the efficiency related to mechanical properties: mercerisation [44,45], maleation treatment [46,47,48], silane treatments [48,49,50], benzoylation treatment [51], peroxide treatment [52] and plasma treatment [53,54]. On the other hand, the study by Ortega et al. [40] identified a growing interest in the use of waste and industrial bioproducts from other processes. Again, as an attempt to lower environmental footprints and lower cost associated with virgin plastics, it was assessed for both dry-blended and melt-compounding material preparation methods. The authors also highlighted the need for life cycle assessment of materials using studies to quantify the overall balance in environmental impact. Reviewers focused on topic areas such as composites with glass fibres or particles [49,54,55,56], nanoparticles such as zinc oxides [57], nanoclay and carbon nanofibres [53,58,59,60,61,62], and finally other fillers reporting on calcium carbonate [63], copper slag waste [64] or residues from mining processes and basalt powders [65,66]. The motivation of this review was to fill the gap for inorganic fillers in sustainable applications for rotational moulding, considering the huge amount of by-products/wastes produced by industrial processes that are not sensitive to temperature and are available in powder form. The authors believed that energy consumption in the processing of composites was unexplored and required further assessment, whilst they also suggested future work assessing the environmental behaviour of materials in application. Yadav et al. [43] reviewed both natural fibres and inorganic filler in their work. The recent review was divided into topics on how the addition of such material affects the matrix, namely, the effect of flow and viscosity, effect of particle size, effect of heating and cooling rate and potential degradation and ageing effects. The authors reviewed each material and concluded that particle loading up to 10 wt% created improvements in impact strength, and treated fibre loading can be increased by up to 30–40% compared to untreated fibre loading [43]. The authors expressed that reduced particle size of the filler/fibre can increase strength according to reports within the literature. Most significantly, from the investigation, Yadav et al. suggest that a critical particle size of around 30 nanometres can provide an increase in the modulus based on the literature. Yada et al. continued to summarise the literature with comments on the importance of antioxidant packages for PE, specifically for maintaining a good impact strength for different processing times [43]. Finally, Sasi Kumar et al. [42] offered a broader review of natural fibres for light weighting for many processing methods like a hand layup, compression, injection, filament, spray and rotational moulding. The authors highlight the use of coir, flax, agave, sisal, pineapple and palmyra fibres whilst discussing the benefits of the RM process. Given that this review has a broader scope, fewer RM data were portrayed. Regarding the only study, by Abhilash et al. [44], as mentioned by Khanna et al. [41], where the authors stated a 10 wt% wood dust addition to LLDPE, an ‘acceptable strength’ was reported on in [42].

In addition to these studies, more publications have been made within this area of research, which were returned as results in the literature search but not mentioned in the previous reviews. All publications are within the past 5 years. Natural fillers such as black tea, giant reed, wood, hemp and rice were amongst findings accompanied by the experimental work with recycled carbon fibres [30,31,32,33,34,67]. Abhilash et al. [34] reported that the addition of risk husk improved the vibrational properties from an experimental modal analysis (EMA), alluding to suitability for automobile vibrating applications by up to 15 wt% addition.

Findings from Ortega et al. reported on the addition of giant reed fibres from invasive plant species, and the dry blending with PE and PLA. The authors claimed that impact performance was acceptable at 5% addition [30]. It was reported that sieving allowed a greater quantity of fibre to be introduced and 20 wt% can be achieved without significant reduction in properties. While not only typical, monolayer composites have been studied recently but also composite foams by Vazquez Fletes et al. [31]. The researchers studied bilayer materials with foaming wood and linear medium-density composites. The authors declared that poor interfacial adhesion reduced impact strength between 47% and 52% with a 10 wt% and 15 wt% addition of wood fibres. Increases in the flexural modulus were shared with agave addition, and further work was suggested to limit gas migration during foaming [31]. The combination of recycled polymers and natural fibres has also been studied, and Arribasplata-Seguin et al. [32] investigated the use of recycled (post-injection moulding processing) high-density polyethylene (rHDPE) from bottle caps from an injection moulding grade and capirona wood particles. This was found to be the only study combining recycled polymers and natural fibres. Accordingly, moulded parts formed well, and similar trends to other literature were observed whereby increasing content impeded the sintering process, thus offering inferior performance for composites compared to the neat rHDPE. Arribasplata-Seguin et al. identified the optimal particle size in the study with some improvements in the tensile modulus as outlined in Table 4. Alternatively, recycled fibres were researched by Oliveira et al., and the authors uncovered that recycled carbon fibres were found to have superior Young’s modulus. There was an increase in performance relative to neat material by 350% in Young’s modulus and a 45% increase in tensile strength when treated with MAPE. Hybridisation mixtures of hemp fibre and recycled carbon fibre at 5 wt% were declared to achieve the same increase in the modulus. It was also reported that Young’s modulus of the hybrid composites increased with a greater addition of the recycled fibre. For example, increasing from 20 to 50% recorded such an effect and the authors stated that this was due to the high stiffness of the carbon fibre and reduced porosity in the final moulded part. Treatment of fibres with matrix acid (nitric acid) was thought to improve adhesion with maleic anhydride grafted polyethylene (MAPE) [33].

The PRISMA search presented some more recent studies from the past 12 months. For example, Ortega et al. [35] reported on the addition of ignimbrite from quarries. The authors found that the addition of the inorganic dust and addition of pressurisation achieved up to 27% time saving in cycle time. The use of pressurisation in the composites produced a similar thermomechanical response to neat PE via a dynamic mechanical analysis (DMA) [35]. The study offers scope for further pressurisation and composite studies given cycle time savings and property enhancement. Studies assessing multilayer composites with banana fibres have been explored using melt compounding and dry blending techniques by Ortega et al. and Kelly-Walley et al. [36,39]. Kelly-Walley et al. reported on the size reduction and changes in the aspect ratio after pulverisation for melt compounded composites [36]. Differences in rheological properties were highlighted, showing a reduction in viscosity due to reduction in fibre size and the aspect ratio, and cycle times were extended due to multi-shot processing. It was found that mechanical properties were inferior to neat polyethylene. Ortega et al. [39] used dry blending to avoid any thermal degradation due to compounding processing, also determining that NaOH pre-treatment of fibres enables an increased performance. In summary, the study claimed that 10 wt% composites did not achieve consolidated parts; it was also reported that PEMA did not offer any increased performance, and a significant increase in cycle time (at least 10 wt% per part in heating cycle) was recorded. More work has been undertaken focusing on the use of banana fibres by Ramkumar et al. [38]. The study focused upon LLDPE/banana fibre composites prepared via dry blending between 5 and 40 wt%. Fibres were prepared by heating to remove moisture and then ‘crushed’ to a fine powder. The researchers concluded that the MFI declines upon increasing the fibre content of the composite. MFI changes from 3.5 g/10 min (5%) to below 1.5 g/10 min (30 wt%). In the study, 10 wt% of the fibre was recommended as the optimal loading range [38].

Hybrid systems have also been explored with the introduction of TiO_2_–lignin. Bula et al. [37] reported that when lignin is used with TiO_2_ in a dual-filler system, higher thermal stability than the lignin alone can be obtained. According to Bula et al., the hybridised system with both fillers enabled the rotomolded containers to exhibit improved compression resistance with slightly lower impact resistance. The compression tests, characterising the load and deflection relationship, found that all composites had a lower minimum-energy-to-crack resistance between 4.5 and 7.5 J compared to the LLDPE achieving 9 J. Mean compression force exceeding LLDPE was achieved by three different composite formulations, while maximum compression values (specimen 1) from 5% TiO_2_–lignin (1:1) exceeded LLPDE by over 70 N. Results were attributed to narrow particle size and low polydispersity of TiO_2_ resulting in reduced agglomeration [37].

Generally, there is significant interest in natural fibres in the RM composite field. This is a potential way to incorporate natural materials and become more resourceful. The next section outlines more of the same, with a greater focus on biopolymers and biocomposites, choosing instead a bio-based or biodegradable matrix. Some recommendations for future research are outlined by the reported literature, for example, the assessment of benefits to energy consumption and processing consumption when utilising fillers and fibres [35]. Finally, the preparation strategies focusing on pre-treatment were a strong theme and the influence on the properties of articles with and without these considerations.

#### 3.2.3. Biopolymers and Biocomposites

In present-day industrial interest and the polymer processing literature, another topic of significant focus is the topic of biopolymers and biomaterials. An area of research observed in the search results was biopolymers and biocomposites where the polymeric matrix is derived from renewable bio-feedstocks rather than fossil-based sources. Benefits of such materials can consist of feedstocks from renewable resources; availability of feedstock; advanced functionalities; potential for biodegradation, thus easing plastic pollution; use for multiple processing methods; and recyclability [68]. Current state-of-the-art biopolymers and biocomposites in rotational moulding are generalised in Table 5.

Some studies retrieved were included in the review by Khanna et al. [41] and were investigating buckwheat and polylactic acid (PLA), PLA and agave fibres and attempts to improve compatibility between natural fibres and green bio-polyethylene [46,47,69].

Aniśko and Barczewski studied bio-based PE and black tea waste from a tea distribution company [67]. Black tea would otherwise have been removed to landfill. However, the tea was of interest due to the antioxidant activity it can provide with catechin extracts; the theaflavins and thearubigins still present in spent tea are able to scavenge radicals and protect the polymer chains [67]. Black tea was found to reduce the carbonyl index (a measurement of the extent of induced oxidation during processing) compared to neat PE. The greatest reduction to 0 from around ~1.7 was reported at 10 wt%. Despite this improvement, further findings from Aniśko and Barczewski included that mechanical strengths were said to have a ‘downward trend’ with increasing filler content. The Young’s modulus decreased by nearly four times at 10 wt% of black tea, for example [67]. In addition, some studies assessed the blending of natural fibres with, specifically, bio-based materials such as PLA/PE in this area. To list a few studies in this area, Robledo-Ortiz et al. assessed natural fibres and green polyethylene biocomposites with Bio-PE/agave composites [46], Barczewski et al. investigated the use of waste copper slag as a filler in PLA [64], PE/PLA with the addition of a husk filler was assessed by Andrzejewski et al. [69] and Perez-Fonseca et al. explored wood/PLA composites, comparing rotational moulding processing and compression moulding [46]. In the case of the publication by Barczewski et al. [64], residue from the filler and the degradation of the polymer showed by TGA was thought to be caused by metal oxides in copper slag and was thought to degrade the PLA matrix by depolymerisation and hydrolytic degradation due to residual water being released [64]. The PLA was almost completely amorphous and the filler addition had negligible nucleating ability. For composites up to 10 wt%, copper slag achieved increased stiffness and hardness. The highest G’ (storage modulus) (at 25 °C and 80 °C) from a thermomechanical analysis and Young’s modulus were recorded for 10 wt%. Furthermore, Robledo-Ortiz et al. [47] explored the combination of PLA and agave fibre for the construction of biocomposites. Modified PLA with maleic anhydride (MA) was used in an attempt to improve adhesion, continuing along the common focus to increase fibre–polymer interaction. According to Robledo-Ortiz et al., the MAPLA (maleic anhydride grafter polylactic acid) layer on the natural fibre increased the effectiveness of stress transfer and mechanical properties. Flexural strength was said to increase and the flexural modulus increased from 2.4 to 3.0 GPa at 10 wt% using MAPLA, and in accordance with other studies, fibre addition significantly reduced properties. Reportedly, impact performance was reduced upon the addition of fibres and with the treatment, the treated fibres had reduced impact strength [47]. The researchers reported that the treated fibres had greater adhesion, resulting in the fracture of the fibre and the matrix, while untreated fibres experienced ‘pull-out’, allowing greater energy dissipation [47]. Similarly, Andrzejewski et al. investigated PLA as a matrix for biocomposites with buckwheat husks (BHs) but also assessed bio-based polylactic acid (PLA) [69]. It was observed that density reduced and porosity increased with increasing filler content, and the authors also found, via a rheological analysis, that viscosity increased for both materials with increasing addition. The former was attributed to PLA degradation and growing hydrodynamic interactions for PE. SEM was interpreted to show a greater interaction between the filler and matrix with only minor interfacial separation compared to full gaps and strong debonding behaviour in PE. A lack of modulus change recorded by Andrzejewski et al. upon tensile testing, was thought to be due to high porosity and PLA/BH composites’ increase in brittle behaviour. Other studies reported on the use of valorised *Ricinus communis* particles in PE and PLA, communicating significant reductions in impact strength when exceeding 5 wt% and reductions in tensile strength in PE, but some increased in PLA [71]. Robledo-Ortiz et al. [46] noticed poor compatibility between bio-based PE and natural fillers. Studies investigated ‘Green-PE’ bio-based agave and core fibres using maleic anhydride (MA) grafted polyethylene (MAPE). Authors applied treatment after drying the fibres by dissolving MAPE in xylene and adding fibres to the solution. Untreated fibres were found to reduce the tensile strength by 45% at 30 wt%; treated fibre addition, however, recorded increases compared to neat PE by up to around 40%. Impact strength was suggested to significantly reduce (50% reduction or more) compared to neat Green-PE; despite this, the treated fibres outperformed non-treated ones [46]. Other efforts to compatibilise natural fibres have been made with PLA as the matrix, specifically further studies from Robledo-Ortiz et al. involving the use of glycidyl methacrylate grafted polylactic acid (GMA-g-PLA) to increase the interfacial adhesion between PLA and agave fibre. This treatment was also reported to reduce water uptake, for example, at 25 wt%, untreated, GMA-treated and twice-GMA-treated fibres had 23.8%, 20% and 15%, respectively. From SEM imaging, it was suspected that this is a result of reduced porosity and improved compatibility, thus explaining the lower moisture absorption [70].

In summary, it appears that the addition of such natural materials is preferred via the dry blending method due to the reduction in thermal exposure to the fibres preventing degradation. Addition above 20 wt% was generally seen to significantly reduce material performance. The use of compatibilisers achieved increases in performance but was not always included in formulating such composites. There was some novel use of fillers to increase thermal stability of a matrix like black tea, for example. Life cycle assessment relative to other materials was not included but was previously mentioned by authors as being beneficial.

### 3.3. Rotational Moulding Process Development

This section focuses on the improvements to be made in the rotomoulding process, mainly on energy consumption and cycle time reductions. This is a key variable in improving process sustainability.

Focusing specifically on rotation speed, and therefore mould speed, Glogowska et al. [73] investigated energy consumption. Moulding linear low-density polyethylene (LLDPE) at various auxiliary and major axis combinations and varying rotation coefficients allowed a full characterisation of the influence of mould speed on energy consumption. The highest energy consumption was reported for a 4:1 ratio and lowest for 1:1. Overall, the authors also stated that the greater the main axis rotation, the higher the energy consumption becomes. The study presented that based on the data published by Glogowska et al., mould speeds could potentially reduce energy consumption and have statistical significance regarding the material performance and part thickness. Another method assessed the ability to reduce energy consumption using micro-active composite materials. Specifically, micro-wave-susceptible inorganic compounds (MWSICs) were of interest with methyl phenyl silicone resin to Luciano et al. [74]. The authors focused on modifying the conventional rotational moulding process. Inorganic compounds such as silicon carbide (SiC), iron (II) silicate (Fe_2_SiO_4_), ferric oxide (Fe_2_O_3_), titanium oxide (TiO_2_) or barium titanate (BaTiO_3_) were investigated. The researchers commented that dielectric heating using microwaves when measuring absorbed power reported savings in time and energy compared to the conventional electric resistance heating process [74]. This was justified by the absorbed power calculated from the dielectric constant of the MWSIC materials [74]. Testing with ISO 527 [75] showed comparable performance from the microwave-assisted process to the classic processing of materials according to Luciano et al.

Continuing with the theme of energy consumption studies carried out, McCourt et al. [76] critically evaluated the environmental, economic and productivity benefits of new industrial technologies. A comparison between conventional and robotic rotational moulding processing machines was performed. According to the authors, the starkest difference between the methods mentioned is that the robotic system presents the ability to heat directly on the rotational moulding tool. McCourt et al. identified that when reaching an identical PIAT, it was found that the electrically heated method was over 14 times more efficient (increasing from 35% to 51%). The authors concluded that time savings of 22% per part were achieved with conductive technology compared to the conventional oven process. Finally, another post-process development was reported on by Tyukanko et al. [77]. Focus was on ensuring that well-processed products were acquired, and the authors investigated three degrees of sintering (under processed, normally processed and degraded material) at varying thicknesses (7.5 mm, 8.5 mm and 9.5 mm) using ultrasonic signals (USSs). It was reported that it is possible to determine the quality of PE sintering through an analysis for a rotationally moulded part by an analysis of the amplitude of the third harmonic of β USS using the mirror–shadow method.

Studies have made progress towards increasing and measuring efficiency and therefore potential modifications to reduce the amount of scrap and energy usage. Considering the progress towards industries of the future, artificial intelligence (AI) and increasing degrees of automation, it is fair to expect the exploration for applying such technologies to RM, as previously mentioned by Crawford and Kearns [14]. There is not one dominant technique in this review, but various methods trialled such as microwave technologies, rotation ratios, ultra-sonic signals and speeds and measuring of efficiencies for conventional and more recent processes.

### 3.4. Modelling and Simulation for Rotational Moulding

The prediction from numerical modelling and simulation with various packages can allow a greater estimation of processing parameters, thus reducing energy expenditure, raw materials and time before optimising the process. Various areas employing such methods to the process method have been explored. The models will not be outlined in detail; only names, the outcome and applications will be shared. For specific model information, publications should be referred to and can be found in the References section.

Chandrasekar et al. [78] developed a data-driven economic model predictive control (EMPC) for the rotational moulding process. The model was developed on data from a uniaxial set-up lab scale machine. The methodology described that measuring impact energy and the ‘sinkhole’ or pinhole area is undertaken before the model is complete by placing them into an EMPC scheme. Details on the model and data can be found within the publication; however, applications of such model were suspected to achieve good product quality and specification-compliant parts while minimising operating costs. The authors stated that the rotational moulding process has non-linearities and is a multi-stage process and due to this, the single model may not be sufficient. It is expected from the authors’ comments that further work will explore a reidentification algorithm for rotational moulding specifically.

Cai et al. [79] focused on coupling the Smoothed Particle Hydrodynamics (SPH) method with the Mohr–Coulomb material methods. The authors reported comparisons to the Discrete Element Method (DEM) numerical benchmark and validated two experimental results. Findings reported the technique to be accurate when the ratio between the SPH particle radius and drum radius was equal to 0.01. Observations were thought to deliver more appropriate methods for an industrial-scale process compared to DEM where the number of particles <20,000 but SPH can be used when millions of particles need to be predicted [79]. This method could offer improvements in understanding the contact of the mould surface with the powder pool, and address benefit challenges such as non-uniform wall thickness, thinning and difficulty covering specific areas.

Seregar et al. focused specifically on warpage simulation [80]. A drawback outlined was the limited data in the literature on temperature-dependant material properties. Despite this, simulation results were found to be within the deviation range of 1.2–21.2% (others at 69%) dependant on processing conditions. The authors suggested that despite some model limitations, this is a major contribution to rotomoulding simulation. Key highlights from the study stated that the cooling rate was directly proportional to the degree of warpage and maximum warpage occurred when the greatest temperature difference was evident between the part and mould, offering practical considerations for rotational moulding when experiencing warpage. Ubene and Mhaskar [81] explored a multiphase model for the rotational moulding process. The authors concluded that a multiphase subspace identification (MPSSID) was introduced with a good degree of success for modelling a three-phase model [81]. Reportedly, the model was tested on pre-existing data from a uniaxial process, and it was found that a three-phase model best predicted the temperature trajectory of the internal air trace, translating into product quality prediction improvements. It was suggested that improvements in more robust model predictive control (MPC) would ease upscaling from laboratory to production scale use.

Modelling of rotational moulding has focused on the heating, cooling and particle movement. Many of the studies have been based upon or validated with lab-style data. Authors suggested that they can be used in a manufacturing environment with some adjustments and further work, which is a positive contribution towards improving process control and prediction and thus the overarching theme of sustainable development.

### 3.5. Sustainable Applications Using Rotational Moulding

This literature review highlighted product development work for applications, which will contribute to a sustainable society. Specifically, search results were found to focus on contributing to the emerging hydrogen economy, with applications directly related to hydrogen storage and type IV hydrogen vessels.

Chashchilov et al. [82] selected the rotational moulding process for prototyping of high-pressure storage cylinders, justified by the benefits of the batch process and the ability to have a small production run. Polyethylene powder was used to produce the liner of the article before glass fibre was wound around the polyethylene rotationally moulded part. The binder composition and binding hardener in the polymer composite material shell were assessed. This example demonstrates the potential use of RM products in the storage of high-pressure gaseous fuels. Motaharinejad et al. [83] investigated hydrogen storage tanks’ metallic connector and how adhesion can be improved between this part and the polymer. In this case, rotational moulding was outlined as preferred over the blow moulding methods as rotational moulding allows the simultaneous manufacturing of the liner and assembling the boss in one process. Blow moulded articles would require an extra unit operation for welding and assembly. The authors assessed the pre-treatment influence on adhesion, and methods adopted were anodising, flaming, sandblasting (at different particle sizes) and PEG (polyethylene grafted) coating. Characterisation techniques optical microscopy and SEM were used to assess typography, and assess mechanical behaviour at the aluminium (Al)/polymer interface. SEM and optical techniques were interpreted to show that sandblasting achieved the greater roughness value and removal of aluminium oxides. Upon the addition of the PEG coating, the authors reported greater adhesion attributed to hydrogen bonds between the grafted carboxyl groups in the PE and hydroxyl groups in the aluminium. Data obtained from the shear test described PEG to have the shear stress of 26.71 ± 0.1 MPa compared to a non-treated sample, which achieved 1.61 ± 0.1 MPa.

It has been demonstrated that developments in hydrogen storage involve using RM as a polymer processing technique for manufacturing. The pre-treatment of the boss and prototyping are the focuses returned within this literature search. In this case, no material development for this application was presented, nor process monitoring. This may be a limitation of the literature review method; however, such areas may be of interest in future publications.

## 4. Conclusions

This review publication outlines many developments in the rotational moulding method, material development, process development, modelling and contributions towards sustainable applications. The wealth of the literature has demonstrated the current position on today’s state of the art.

- The current state-of-the-art rotational moulding literature contributing to sustainability undertaken with an approach comparable to the PRISMA literature review method heavily focused upon materials and composite characterisation and assessment. From retrieved studies, 75% were categorised as materials, 9% as the process, 9% as the process and modelling and 4 as sustainable applications. The introduction of further search engines, which includes non-academic publications, such as Google Scholar, might be beneficial for future reviews; similarly, the introduction of conference papers would add some interesting insights. However, this was not addressed within this work due to the difficulties in obtaining the full papers for such works, as they are not usually easily accessible.
- Recycling appears to be increasingly an area of interest in rotomoulding, as in with a constant growth, like for many other processing methods. The research work performed so far highlights the challenges with viscosity, degradation and impact performance when using polyethylene, including use in blends. A limitation of studies reviewed in this area is the low quantity of studies, from which there is still less focus on polyethylene specifically. This makes it challenging to conclude on the general trends between rotational moulding and recyclate polyethylene adoptions.
- Few publications adopted natural fibres and recyclate material simultaneously. There was also limited cross-over between the other subsections. Potentially, in future work, once each research area grows with greater published data and increased material usage in industry, this will facilitate the modelling and simulation of more novel materials such as biocomposites, biopolymers and recyclate. This also highlights the relevance of this literature review performed and the need to perform further work to solve this gap.
- Fibre/filler research was seen to be heavily dominated by natural fibres and fillers. A keen research interest was evident with four reviews recently published in this area. The use of biomaterials and biocomposites reported the use of waste materials providing increased thermal stability; this offers some novel findings, which could offer scope for further research.
- As mentioned by authors, material-based research and process modifications would benefit from life cycle assessment (LCA), or an indication as to what degree a reduction in a carbon footprint would be achieved as a result of the work. This would enable a clearer consideration of the potential for progress towards sustainability and the current limitations.
- Studying sustainable applications like those supporting the hydrogen economy is new and emerging. Only two returns were made, which may highlight a limitation in the review method. Such limitations could be addressed by collecting further studies and reviewing this area individually, allowing stronger conclusions on the connection of hydrogen and rotomoulding. Developments for future use are expected to continue in the hydrogen economy, as well as a continuation in other prospects such as the automotive sector, tanks and leisure with a shift towards materials of a sustainable nature, and developments in each sector can benefit from more sophisticated technologies and automation.

## Figures and Tables

**Table 1:** Number of results retrieved from Scopus based on keywords.

Keywords	Scopus Results
Sustainab*	20
Recyc*	32
Bio*	58
Natural	51
Waste	26
Energy	52
Simulation	38
Modelling	44

**Table 2:** Categories of literature review based on the search returns from Scopus systematic review.

Categories	Sub-Category	Publications Appearing in This Review
Material Research	Use of Waste and Recyclate	9
	Natural Fibres and Fillers	14
	Biopolymers and Biocomposites	8
Process Developments		4
Modelling and Simulation		4
Developments for Sustainable Applications		2

**Table 3:** Summary of Recyclate Literature.

Refs.	Virgin Material	Recyclate/Recycled Material	Percentages Assessed	Conclusions
[19]	General Purpose LLDPE (Linear Low-Density Polyethylene)	Post-consumer plastic waste (PCR) Reground tanks (RGTs) (simulated recycling of RM grade)	100 wt%	Recyclate blends have a very larger increase in viscosity within the low-shear region Traces of PP (polypropylene) identified in PE recyclate Powder quality issues were found when cryogenically grinding blends, resulting in direct impact on sintering, densification and mechanical properties Recyclate blends had total impact results below 1 J/mm, while vPE was at 7.24–17.01 J/mm
[20]	MDPE (Medium-Density Polyethylene) (vPE)	Recycled HDPE from (1) bottles, (2) pipes and (3) household waste	50 wt% vPE/50 wt% rHDPE	Peak impact energy of vPE was at 7.9 J/mm, and some recyclate blends achieved up to 9.3 J/mm for compression-plaque moulded plaques. An 85–87% reduction compared to MDPE was recorded in rotomoulded samples A 20–30% decrease in flexural modulus with all recyclate blends PP in some rHDPE determined by DSC
[21]	MDPE (vPE)	Residual polymers from cable waste with 1–2 wt%	0–50 wt% Cable Waste	Maximum 50% residue could be processed by RM Fractions up to 35% can be incorporated without important reductions Elongation was most affected after just 10% addition Impact strongly reduced with addition
[22]	LLDPE	rHDPE (recycled high-density polyethylene) from blown bags	0–50 wt% Blends and 100 wt% rHDPE	As HDPE content increased, tensile strength decreased LLDPE had a lower impact strength compared to recycled HDPE Good phase balance observed in SEM
[23]	N/A (no virgin material used in this study)	Post-consumer PP and HDPE (high-density polyethylene) from water and alcohol HDPE bottles	100 wt%	Useful for construction application UL-94 V0 achieved with addition of 5 wt% alumina HDPE block would be an excellent material for modular construction
[24]	vLDPE/Foam Structure	Reprocessed LDPE/foam structure	0 wt% and 100 wt%	Crack formation at 30 J for reprocessed structure; however, virgin structures resist all crack formations Reprocessing reduced residual strength due to degradation
[25]	N/A	PLA (polylactic acid) disposable cups	100 wt%	Successful parts produced Annealing of the PLA cups before milling and the extrusion increased the crystallinity of material Properties linked to polymer structure
[26]	LDPE (vPE)	Ground tyre rubber (GTR) in regenerated (RR) and non-regenerated form (NRR)	0 and 20 wt%, 35% and 50 wt% GTR	Prepared by dry blending and melt compounding A 0–20 wt% homogenous rubber phase was observed The authors observed 20–50 wt% greater voids Properties decreased with increasing addition due to poor adhesion at GTR and LDPE interface.
[27]	N/A	rHDPE	100%	rHDPE was successfully used in foams via dry blending for RM Average cell diameter increased with increasing CBA content

**Table 4:** Summary of all filler/fibre literature reviewed.

Refs.	Resin	Fibre	Conclusions
[30]	LLDPE	Giant Reed (*arundo donax*, *pennistem setaceum*)	Impact properties greatly affected by presence of fibres, 5 wt% said to give acceptable impact strength Sieving can help to increase loading rate without reducing mechanical performance Fibre size demonstrated to have significant influence on composite performance
[31]	LMDPE (low–medium-density polyethylene)	Agave Fibre	Thickness of each layer increased with increasing agave addition and blowing agent Density reduced from 0.93 g/cm^3^ to 0.44 g·cm^3^ (55% reduction) Increase in Young’s modulus by 23% and reduction in tensile strength of 13% with introduction of fibre Impact strength reduced up to 52% with fibre addition
[32]	HDPER (unused white bottle caps)	Capirona Wood Particles (CWPs) from Capirona Tree (*calycophyllum spruceanum*) Pine Wood Particles (PWPs) from Pine Trees (*pinus radiata*)	Determined that parameters that influence the sintering process and allow convenient performance were (a) 15% wood particles, (b) wood particle size of 297 μm and 500 μm and some defined processing conditions CWPs and PWPs in relation to the above achieved tensile strength of 17 MPa and 18 MPa, respectively. As a result, up to 17% reductions in tensile strength were compared to HDPER and there were up to 16% increases in elastic modulus
[33]	LMDPE MAPE (maleic anhydride polyethylene)	Hemp Fibre (*Cannabis sativa*) Recycled Carbon Fibre (RCF)	Recycled carbon fibre treatment with nitric acid up to 120 min was beneficial for increasing oxygen groups No significant increase in performance was achieved in the rotational moulding RCF composites with pre-treatment, and is therefore not advantageous RCF composites had superior Young’s modulus to PE regardless of pre-treatment Hybridisation of RCF and hemp reported superior tensile strength and Young’s modulus compared to neat PE
[34]	HDPE	Rice Husk	At 10 wt%, increases in impact (30.5 kJ/m^2^ to 36.8 kJ/m^2^), flexural strength (65.1%) and tensile strength (15.1 MPa to 15.8 MPa) and hardness were observed Failure analysis highlighted that fracture occurred due to debonding and voids were the cause of this debonding
[35]	LDPE (low-density polyethylene)	Ignimbrite Dust (ID)	A 10% reduction achievable with PE using pressurisation Addition of 10 wt% ID showed up to 27% reduction in oven time Thermomechanical analysis showed near-zero adhesion factor at 10% dust loading and reinforcing effect at temperatures over 20 °C
[36]	mLLDPE (metallocene LLDPE)	Banana Fibre	Multilayer/multi-shot approach increased cycle times up to 32% A 65–83% reduction in impact strength recorded compared to neat PE Use of neat PE on external layer provided greatest composite performance
[37]	LLDPE	Lignin and Titanium Dioxide (TiO_2_)	Lignin fillers experienced thermal degradation during processing Addition of lignin reduces impact strength relative to LLDPE Lignin and TiO_2_ in dual system increased thermal resistance, improved compression and reduced impact relative to only lignin
[38]	LLDPE	Banana Fibre (BF)	MFI reduced upon BF loading from 3.5 g/10 min (5%) to 3.022 g/10 min (15 wt%) Up to 10 wt% BF loading achieves satisfactory water absorption
[39]	mLDPE (metallocene low-density polyethylene)	Abaca	Tensile modulus increases only in 2-layer structures at 10 wt%, remaining unchanged for single- and 3-layered parts Thermomechanical behaviour of the composites does not show any significant differences, regardless of the number of layers or the weight of fibre used

**Table 5:** Summary of Biopolymer and Biocomposite literature results.

Refs.	Materials	Properties
[69]	LDPE, PLA Buckwheat (BW)	Density of all PE/BH composites was lower than pure PE. Increasing porosity with increasing BH Highlighted that potential applications could likely be consumer products, furniture accessories and garden equipment
[67]	Bio-LDPE Black Tea Waste (BTW)	Adding 10 wt% BTW prevents thermoxidation of LLDPE, assessed by carbonyl index and rheological isothermal assessment BTW has the functionality to terminate free radicals (good—complete)
[46]	Bio-LLDPE MAPE and Agave and Coir Fibres	Fibres decreased tensile strength of the bio-LLDPE due to poor fibre–matrix adhesion Reduction from 13.7 MPa to 7.4 MPa at 30% coir or agave fibre content Increase to 11 MPa and 15.3 MPa for tensile strength when fibres were treated with maleic anhydride, for agave and coir, respectively Treated coir fibre achieved impact of 117 J/m similar to commercial petroleum-based PE (121 J/m)
[64]	PLA and Copper Slag (CS)	Unmodified inorganic waste was said to achieve a uniform composite structure, especially at lower filler loading Copper slag presence did not significantly affect melting behaviour and crystallin structure Thermal stability did not deteriorate with addition of copper slag Increased stiffness and hardness was achieved at 10 wt% addition. Composites with up to 35% copper slag addition can be used for less demanding applications
[70]	PLA and Glycidyl Methacrylate (GMA) Dicumyl Peroxide (DCP) and N,N-Dimethylformamide (DMF) Agave Fibres	Applying chemical treatment using GMA-g-PLA, fibre–matrix interfacial adhesion was improved and porosity was reduced At 25 wt% with fibres treated twice, improvements were recorded in flexural and tensile properties compared to neat PE
[71]	PE, PLA Ricinus Cummunis Particles	Treated fibres produced thicker walls with a greater degree of porosity and therefore poor mechanical properties. More compact and darker parts were achieved with untreated fibres Excluding 5% addition of Ricinus particles significantly affected impact properties Tensile strength was lower in all composites compared to PE, most significantly at 10 wrt% Signs of accelerated biodegradation for PLA-based composites with Ricinus but not visible by FTIR, but clear on DSC analysis
[72]	Bio-HDPE and Basalt Fibres (BFs)	Dry blending recorded migration of fibres towards a far distance from the mould wall, while melt compounding produced micrometric powders, reducing aspect ratio No significant increases in modulus recorded, attributed to uneven distribution not achieving reinforcement in the composite
